# Supplementary material for: Highly tensile-strained Ge/InAlAs nanocomposites
Source: Nat Commun. 2017 Jan 27;8:14204. doi: 10.1038/ncomms14204 (PMC5290139; doi:10.1038/ncomms14204)
Supplement: Supplementary Information — Supplementary Figures, Supplementary Notes and Supplementary References. [file ncomms14204-s1.pdf]

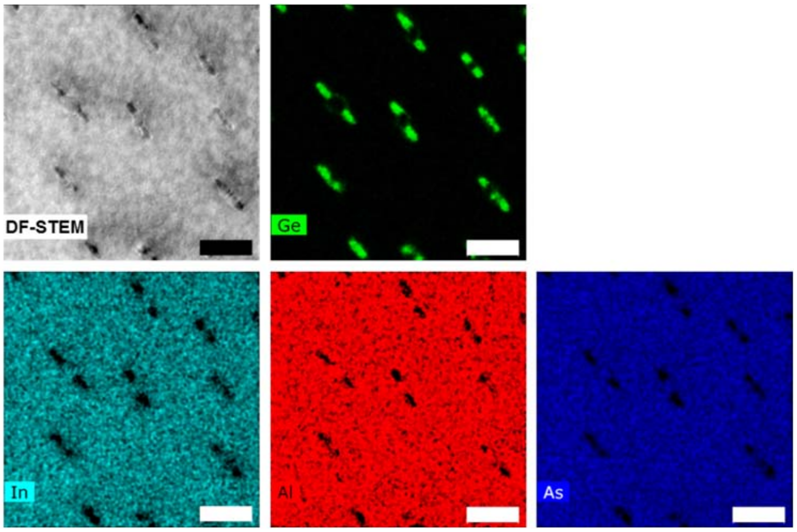

Supplementary Figure 1. Planar-view annular dark-field scanning transmission electron microscopy (ADF-STEM) and energy-dispersive X-ray (EDX) images on a 3.6% Ge/InAlAs nanocomposite grown at 0.2 μm per hour. Many of the Ge nanostructures are branched. The scale bars are 30 nm.

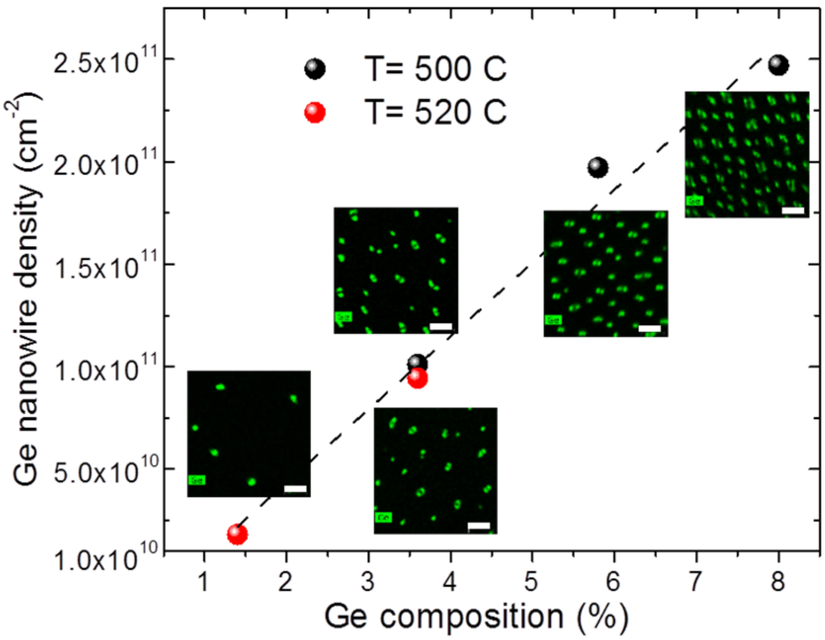

Supplementary Figure 2. Ge nanowire density versus Ge composition. All samples are grown at 1 µm per hour. The scale bars of the inset Ge EDX maps are 30 nm.

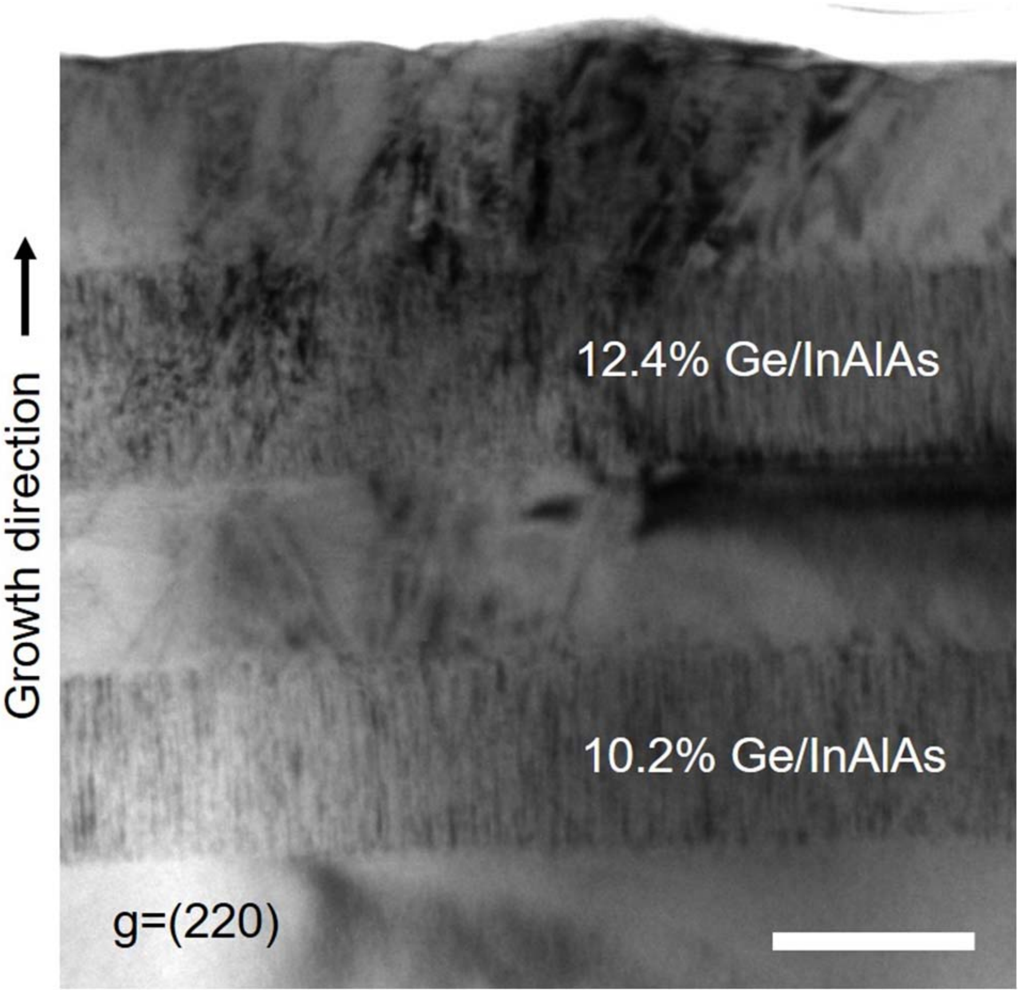

Supplementary Figure 3. Cross sectional bright-Field (BF)-TEM image on 12.4% and 10.2% Ge/InAlAs nanocomposites. 12.4% Ge/InAlAs nanocomposite clearly shows formation of extended defects. Scale bar is 200 nm.

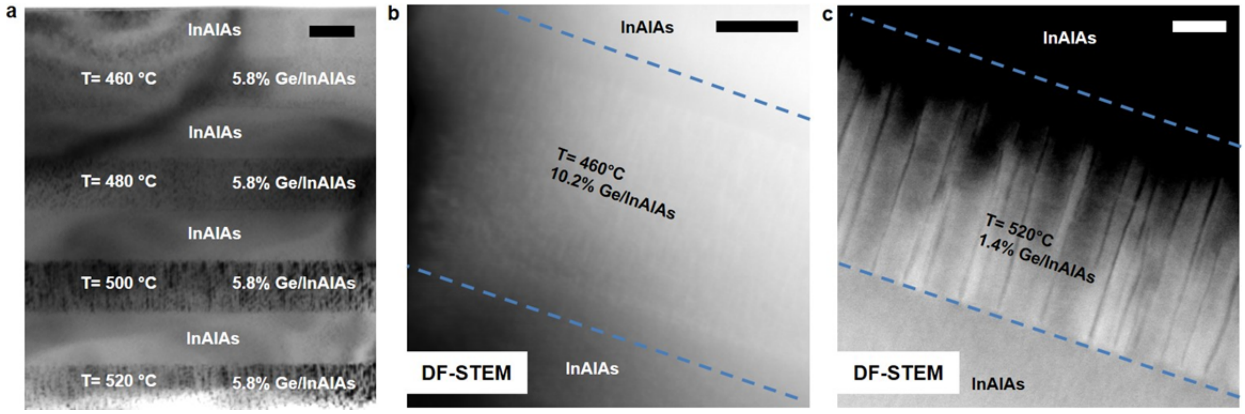

Supplementary Figure 4. Effect of growth temperature and Ge composition. **a**, Cross-sectional BF-TEM with g=(220) shows a sample with 5.8% Ge/InAlAs layers grown at different growth temperature. Scale bar is 200 nm. **b**, ADF-STEM image shows a 10.2% Ge/InAlAs metastable alloy layer grown at T=460 °C. Scale bar is 60 nm. **c**, ADF-STEM image reveals that a 1.4% Ge/InAlAs layer grown at T=520 °C contains Ge nanowires embedded in the InAlAs matrix. Scale bar is 60 nm.

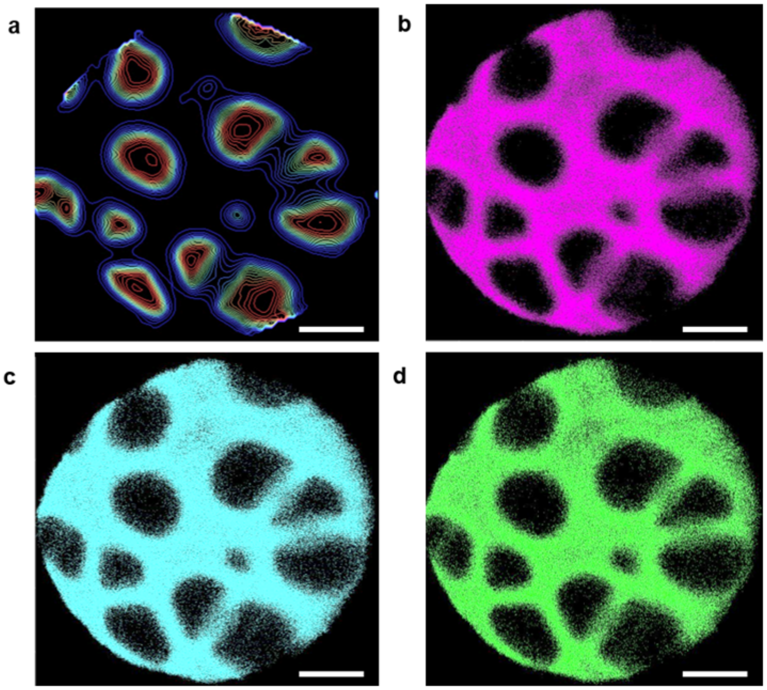

Supplementary Figure 5. Atom probe tomography (APT) transects on 3.6% Ge/InAlAs nanocomposite at 0.2 μm per hour and 500 °C. a, Ge concentration iso-contour transect. **b**, In atoms represented by magenta dots (100 pct). **c,** Al atoms represented by cyan dots (100 pct). **d**, As atoms represented by green dots (100 pct). Scale bar is 20 nm.

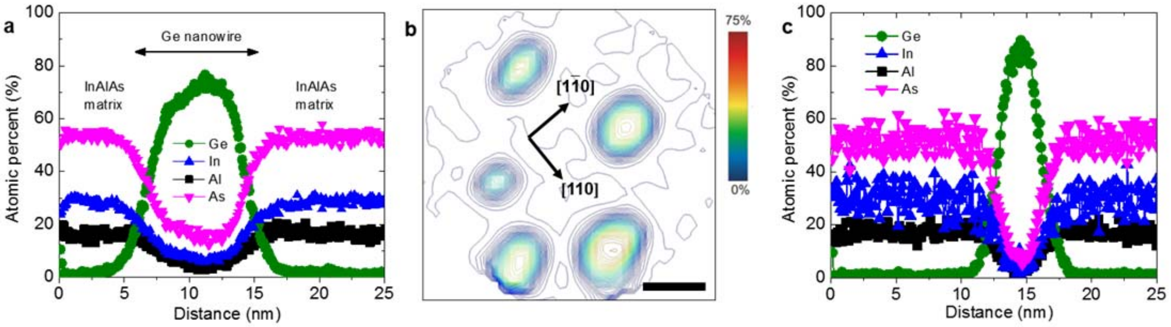

Supplementary Figure 6. EDX line scan and APT transect on 3.6% Ge/InAlAs nanocomposite. a, EDX linescans of Ge, In, Al, and As and **b,** APT Ge concentration iso-contour transect of a 15 nm thick slice of the Ge/InAlAs nanocomposite grown at 1 μm per hour and 500 °C. Scale bar is 10 nm. **c,** EDX linescans of Ge, In, Al, and As from a nanocomposite grown at 1 μm per hour and 520 °C.

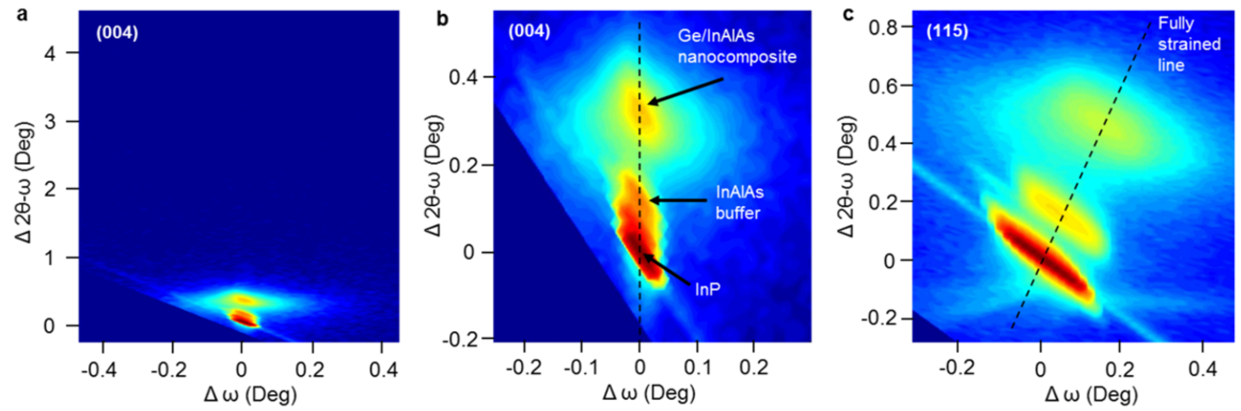

Supplementary Figure 7. X-ray (004) reciprocal space map on 3.6% Ge/InAlAs nanocomposite. a, Wide-angle RSM shows no film tilt in the Ge/InAlAs nanocomposite. **b,** Zoom-in (004) RSM shows that the Ge/InAlAs nanocomposite peak has linewidth broadening. The strained Ge nanostructures induce spread in the InAlAs matrix lattice constant. **c,** (115) RSM reveals that the two epi-layers are fully strained to the InP substrate. The calculated lattice constants of the Ge/InAlAs nanocomposite are 5.868 Å and 5.850 Å for in-plane and out-of-plane, respectively.

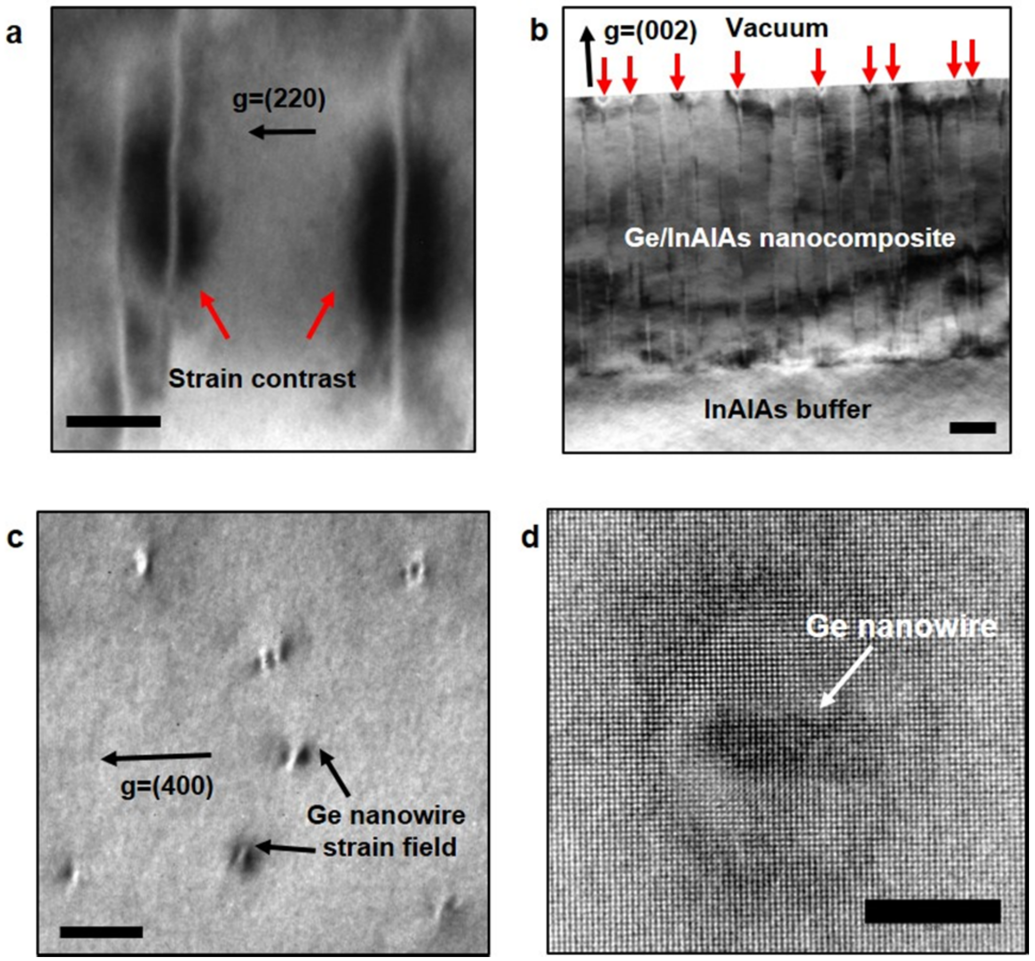

Supplementary Figure 8. Cross-sectional and planar-view TEM. a, Strain-induced contrasts surrounding Ge nanowires with g=(220) (cross-sectional BF-TEM). Scale bar is 30 nm. **b,** Half-circular, strain-induced contrasts indicated by red arrows show the change of the strain state of the Ge nanowires near the surface, g=(002) (cross-sectional BF-TEM); the Ge nanostructures relax due to the free surface. Scale bar is 50 nm. **c,** Strain field around the Ge nanowires is observed from planar-view BF-TEM as well. Scale bar is 30 nm. **d,** Aberration-corrected planar-view Low-angle ADF-STEM image on a single nanowire surrounded by strain contrasts. The scale bar is 5 nm.

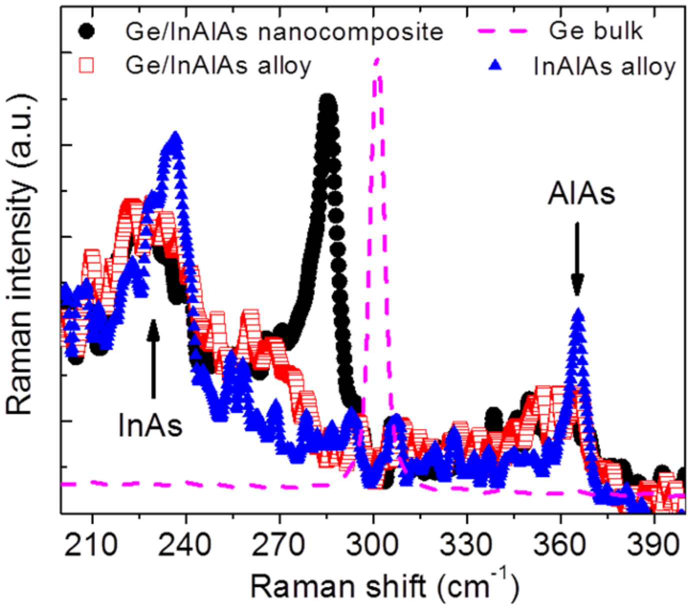

Supplementary Figure 9. Raman spectroscopy in backscattering configuration using a 532 nm laser. Ge bulk (magenta dash), InAlAs alloy (blue solid triangle), Ge/InAlAs nanocomposite (black solid circle), and Ge/InAlAs metastable alloy (red empty square).

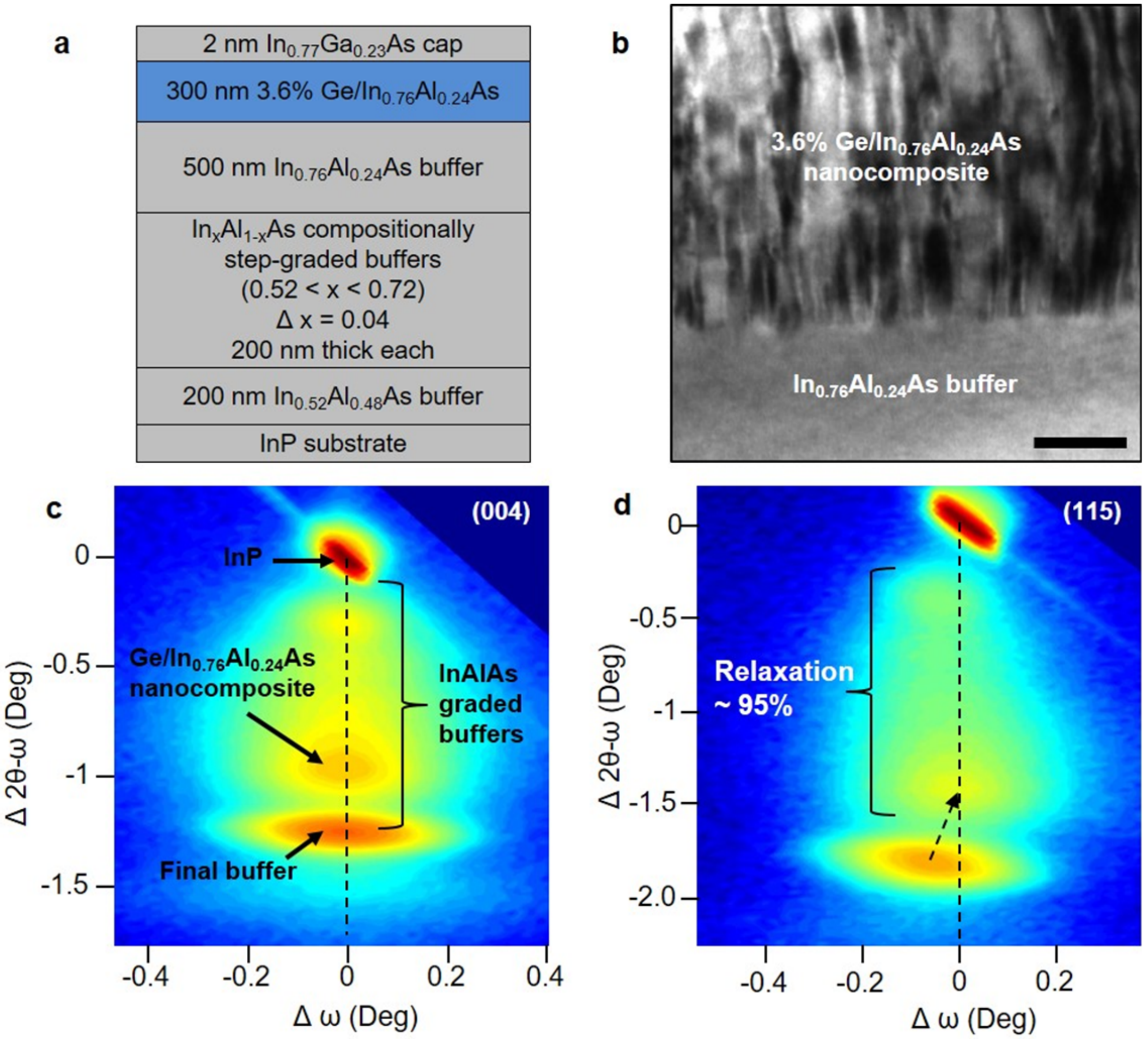

Supplementary Figure 10. A 3.6% Ge/$In_{0.76}Al_{0.24}As$ nanocomposite grown on $In_xAl_{1-x}As$ compositional graded buffers. a, Schematic of sample structure. The $In_xAl_{1-x}As$ graded buffers were grown before the Ge/$In_{0.76}Al_{0.24}As$ nanocomposite film. **b,** Cross-sectional BF-TEM displays phase-separated Ge nanostructures in the $In_{0.76}Al_{0.24}As$ matrix. Scale bar is 50 nm. **c,** X-ray RSM (004) reflection. **d,** RSM on (115) reflection.

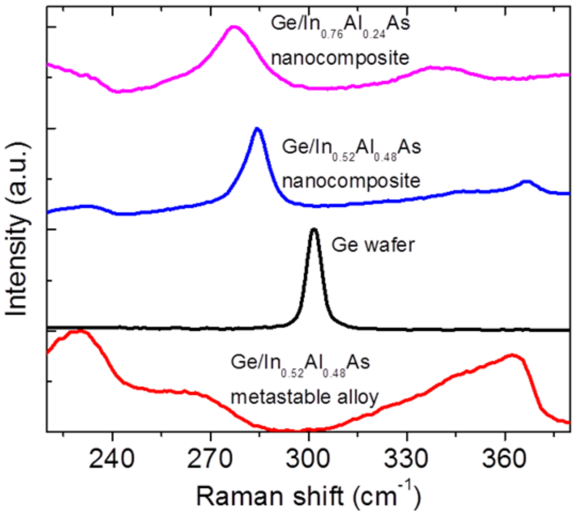

Supplementary Figure 11. Raman spectroscopy in back-scattering configuration using a 633 nm laser. Raman spectra of Ge/$In_{0.52}Al_{0.48}As$ metastable alloy (red), Ge wafer (black), Ge/$In_{0.52}Al_{0.48}As$ nanocomposite (blue), and Ge/$In_{0.76}Al_{0.24}As$ nanocomposite (magenta). Clear Raman shifts of Ge-Ge peaks are observed, showing tensile strain in the Ge nanostructures.

Fd

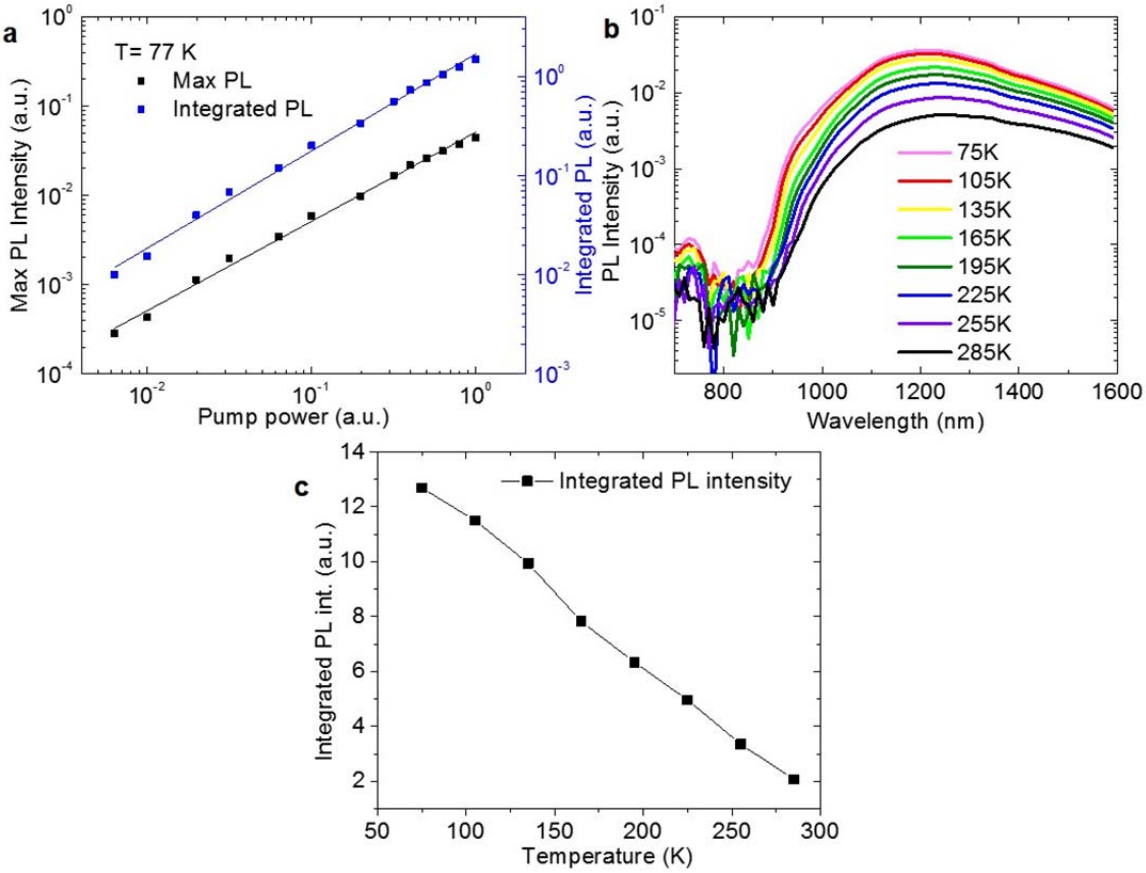

Supplementary Figure 12. Low temperature excitation-dependent photoluminescence (PL) and temperature-dependent PL. a, Peak PL intensity vs laser pump power (blue) and integrated PL intensity vs laser pump power (black), **b,** Evolution of PL spectra vs temperature, **c**. Integrated PL intensity vs temperature (K).

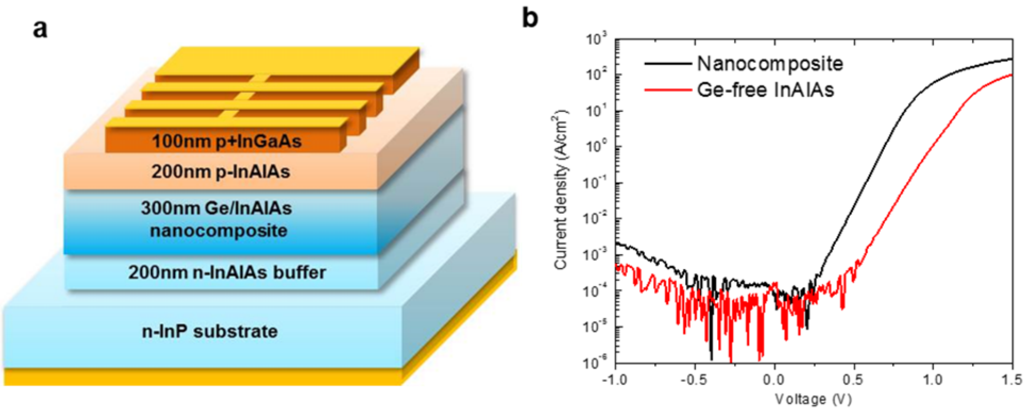

Supplementary Figure 13. Schematic of p-n light-emitting diode (LED) using Ge/InAlAs nanocomposite and I-V curves. a, Standard photolithography and wet-etching techniques were used to fabricate the LEDs. For a control p-n diode, the Ge/InAlAs nanocomposite layer was replaced by n-InAlAs with nominal doping concentration of 2×10^{18} cm^{-3}. **b**, I-V curves in a semi-log plot.

Supplementary Note 1. Ge/$In_{0.52}Al_{0.48}As$ (Ge/InAlAs) nanocomposite growth and structural characterization

Ge/InAlAs nanocomposites were grown as a 300 nm $Ge_x/(InAlAs)_{1-x}$ ($0.014 < x < 0.102$) film on (001) InP substrates at various growth rates from 0.1 to 1.0 μm per hour in a molecular beam epitaxy (MBE) chamber. A 2 nm lattice-matched $In_{0.53}Ga_{0.47}As$ cap was deposited to prevent oxidation. The planar-view annular dark-field (DF) scanning transmission electron microscopy (STEM) and energy dispersive x-ray spectroscopy (EDX) maps of a 3.6 % Ge/InAlAs nanocomposite sample (Supplementary Figure 1) show that dark spots from the DF-STEM image match the location of Ge-rich regions. On the contrary, the elements of the InAlAs matrix are sparse where the Ge nanowires exist. The Ge nanowires extend along the in-plane $[1\bar{1}0]$ direction, suggesting that Ge adatoms have anisotropic surface diffusion length on the InAlAs(001) surface, similar to Ga adatoms in GaAs growth[1]. Further elongation incurs branching for some of the nanowires in the $[1\bar{1}0]$ direction, which was also observed in the ErAs/GaAs[2] and ErSb/GaSb nanocomposite systems[3].

Supplementary Figure 2 illustrates that increasing the Ge composition increases the Ge nanowire density, while the size is nearly constant at both T= 500 °C and T= 520 °C.

Figure 1(e) of the manuscript shows that nanocomposites with relatively low Ge content are free of dislocations and stacking faults, indicating a lack of plastic relaxation. However, for Ge/InAlAs nanocomposites with relatively high Ge content (~12.4%), we observed extended defects as seen in Supplementary Figure 3.

We systematically investigated the growth conditions for Ge/InAlAs nanocomposites by varying the Ge compositions and growth temperatures. Supplementary Figure 4a shows a bright-field (BF) TEM image of a sample consisting of 5.8% Ge/InAlAs nanocomposite layers grown at different growth temperatures, separated from each other by pure InAlAs layers. The 5.8% Ge/InAlAs layers grown at T= 500 °C and 520 °C have phase-separated Ge nanostructures embedded in the InAlAs matrix. However, the layers grown at 480 °C and 460 °C do not form Ge nanostructures and remain as metastable alloys. Two other examples are also shown in Supplementary Figure 4b-c. The DF-STEM image of Supplementary Figure 4b shows that the low growth temperature of 460 °C suppresses Ge phase separation in a film that contains a relatively high Ge composition of 10.2%. In contrast, Supplementary Figure 4c reveals that the high growth temperature of 520 °C drives strong Ge phase separation in a Ge/InAlAs film that contains only 1.4% Ge.

Supplementary Figure 5 shows APT images from a Ge/InAlAs nanocomposite grown at 0.2 μm per hour and 500 °C, clearly showing a lack of In, Al, and As elements where Ge nanowires exist.

EDX linescans in Supplementary Figure 6a reveal that the Ge concentration of nanowires grown at 1 μm per hour and 500 °C peaks at ~75%. The atom probe tomography (APT) image in Supplementary Figure 6b shows ~75% Ge in the nanowires, consistent with the EDX linescans. We have also found that nanowires grown at 1.0 μm per hour but 20 °C hotter exhibit peak Ge concentrations of ~90%, due to the thermally activated nature of surface diffusion (Supplementary Figure 6c).

Supplementary Note 2. Strain analysis of Ge/InAlAs nanocomposite

Biaxial tensile strain for growth along a [100] orientation is theoretically predicted to be more favorable than [110] or [111] orientations for converting Ge into a direct-gap material, since it drops E_g_Gamma more strongly than E_g_L[4]. In the case of a strained, single-phase thin film on a substrate, only the lateral strain component and out-of-plane strain caused by the Poisson effect need to be considered. However, nanocomposites with strained columnar structures enclosed in a lattice-mismatched matrix possess a vertical strain component as well,[5] and this is also observed in our Ge/InAlAs nanocomposites. The X-ray reciprocal space maps (RSMs) on a 3.6% Ge/InAlAs nanocomposite sample reveal three intensity peaks, InP substrate, InAlAs buffer, and Ge/InAlAs nanocomposite (Supplementary Figure 7). The in-plane lattice constants (a_{para}) of the InAlAs buffer and Ge/InAlAs nanocomposite are identical at a_{para}= 5.868 Å, and are fully strained to InP substrate. A relaxed Ge peak is not observed (~ 3° in Δ 2θ-ω) in the wide-angle (004) RSM (Supplementary Figure 7a), indicating that the Ge nanostructures are vertically strained to the InAlAs matrix. The increased linewidth of the Ge/InAlAs film peak (Supplementary Figure 7b-c) is due to the inhomogeneous strain and spatially varying spread in the InAlAs matrix lattice constant.

The main text supports the presence of biaxial tension (i.e. lateral strain) in the Ge nanostructures near the surface using atomic force microscopy and Raman spectra. The cross-sectional g= (220) BF-TEM image in Supplementary Figure 8a shows strain-induced contrast surrounding the Ge nanowires and the lack of plastic strain relaxation at the interfaces. The g= (002) BF-TEM image in Supplementary Figure 8b reveals intense strain fields where each nanowire intersects the surface, which is also consistent with a change in local strain state due to relaxation at the free surface. Clear strain fields are observed around the Ge nanowires from the planar-view BF-TEM image (Supplementary Figure 8c) and from the LAADF-STEM image (Supplementary Figure 8d).

Supplementary Figure 9 shows Raman spectra on various samples by a Horiba-Jobin Yvon HR-800 Raman Microscope equipped with a 532 nm laser. The Ge wafer shows a longitudinal optical (LO) phonon peak at 301 cm^{-1} and the pure InAlAs alloy sample shows InAs LO at ~230 cm^{-1} and AlAs LO at ~365 cm^{-1}. The nanocomposite sample reveals a sharp Ge LO peak at 285 cm^{-1} in addition to the InAs LO peak at ~230 cm^{-1} and AlAs peak at ~365 cm^{-1}. On the contrary, the Ge/InAlAs alloy sample does not have the Ge LO peak while showing InAs and AlAs peaks. The penetration depth of the 532 nm laser in Ge is ~20 nm. Therefore, the Raman spectroscopy probes the Ge nanostructures near the surface only. The 16 cm^{-1} Ge Raman shift corresponds to a 3.64% biaxial tensile strain from equation, $\Delta\omega = b \cdot$ biaxial-tension (b is a phonon strain shift coefficient and we adopt b= -440). Consistent with the Raman spectra taken with the 633 nm laser, the calculated strain value is very close to the lattice mismatch of 3.72%.

We eliminated heating as a possible cause of the observed red-shift by performing the Raman measurements at low incident laser power, specifically for this purpose. Moreover, since the Ge nanowires are embedded in an InAlAs matrix, the little heat that might have generated can be quickly dissipated away. We rule out phonon confinement because, for unstrained freely dispersed Ge nanowires, the observed confinement-induced red shift is very small compared to what we observed in our experiments[6]. Furthermore, the interfaces between the InAlAs matrix and Ge nanowires are coherent, meaning that the two lattices are coupled and phonons can propagate from nanowires to matrix and vice versa, minimizing further the chances of phonon confinement. Phonon confinement due to Ge/InAlAs mass periodicity is also eliminated since confinement in such structures are only observable at very low temperatures[7, 8].

Supplementary Note 3. Ge/$In_{0.76}Al_{0.24}As$ nanocomposite growth and characterization

To demonstrate strain tuning in Ge/InAlAs nanocomposites, we grew a Ge/$In_{0.76}Al_{0.24}As$ nanocomposite layer on an $In_xAl_{1-x}As$ ($0.52 < x < 0.76$) compositionally graded buffer (Supplementary Figure 10a). The cross-sectional TEM image in Supplementary Figure 10b reveals columnar Ge-rich regions embedded in the $In_{0.76}Al_{0.24}As$ matrix. The RSM on (004) reflection in Supplementary Figure 10c reveals no tilt in the epitaxial films (the InAlAs graded buffers and nanocomposite layer). The RSM on (115) reflection in Supplementary Figure 10d shows 85 - 95% relaxation of the InAlAs graded buffer layers. In contrast, the Ge/$In_{0.76}Al_{0.24}As$ nanocomposite layer is strained to the final buffer layer as indicated by arrow (Supplementary Figure 10d). The calculated a_{para} for the final buffer is 5.948 Å and the corresponding in-plane mismatch to Ge is 5.15%, which is very close to the biaxial strain value of 5.3% from Raman spectroscopy as described more in the next paragraph. The out-of-plane lattice-constant (a_{perp}) of the final buffer is 5.973 Å while that of the nanocomposite is 5.949 Å, indicating a 0.024 Å decrease in a_{perp} due to the embedded Ge nanostructures.

The Raman spectroscopy in Supplementary Figure 11 shows a more highly red-shifted Raman peak from the Ge/$In_{0.76}Al_{0.24}As$ nanocomposite sample. The Raman peak at 278 cm^{-1} corresponds to 5.3% of biaxial tension in Ge using the equation given in the main text. The linewidth broadening of the Ge-Ge peak might be due to threading dislocations inherited from the $In_{0.76}Al_{.0.24}As$ graded buffer. We also found from the new $In_{0.76}Al_{0.24}As$ matrix that the AlAs LO peak is red-shifted from ~365 cm^{-1} to 340 cm^{-1} while the InAs LO peak stays at ~230 cm^{-1}, consistent with an earlier study on the Raman spectra of In-rich InAlAs alloys[9].

Supplementary Note 4. Low temperature photoluminescence (PL)

We performed low-temperature (T= 77 K) excitation-dependent PL (Supplementary Figure 12a) and found that both the peak intensity and integrated intensity vary linearly with the excitation density; a linear dependence of integrated PL intensity on pump power is extremely common in studies of novel optoelectronic materials, including InN[10] and InGaAsN[11]. The sub-quadratic relationship may be due to exciton recombination in the Ge nanostructures[12] or loss of carriers to the non-radiative trap states at the Ge and InAlAs interfaces[13]. The temperature-dependent PL measurements in Supplementary Figure 12b-c display an integrated PL intensity increase and a blue-shift of the PL peaks as temperature decreases. Note that 0.22% tensile-strained n-type Ge (an indirect bandgap material) showed decreasing direct bandgap PL intensities at decreased temperatures[14].

Supplementary References

1. Kley A, Ruggerone P, Scheffler M. Novel diffusion mechanism on the GaAs(001) surface: The role of adatom-dimer interaction. *Phys Rev Lett* **79**, 5278-5281 (1997).

2. Singer KE, Rutter P, Peaker AR, Wright AC. Self-Organizing Growth of Erbium Arsenide Quantum Dots and Wires in Gallium-Arsenide by Molecular-Beam Epitaxy. *Appl Phys Lett* **64**, 707-709 (1994).

3. Kawasaki JK, Schultz BD, Lu H, Gossard AC, Palmstrom CJ. Surface-mediated tunable self-assembly of single crystal semimetallic ErSb/GaSb nanocomposite structures. *Nano Lett* **13**, 2895-2901 (2013).

4. Chang GE, Cheng HH. Optical gain of germanium infrared lasers on different crystal orientations. *J Phys D Appl Phys* **46**, (2013).

5. MacManus-Driscoll JL, *et al.* Strain control and spontaneous phase ordering in vertical nanocomposite heteroepitaxial thin films. *Nat Mater* **7**, 314-320 (2008).

6. Wang X, Shakouri A, Yu B, Sun XH, Meyyappan M. Study of phonon modes in germanium nanowires. *J Appl Phys* **102**, (2007).

7. Sood AK, Menendez J, Cardona M, Ploog K. Resonance Raman-Scattering by Confined Lo and to Phonons in Gaas-Alas Superlattices. *Phys Rev Lett* **54**, 2111-2114 (1985).

8. Spitzer J, *et al.* Raman-Scattering by Optical Phonons in Isotopic (70)(Ge)(N)(74)(Ge)(N) Superlattices. *Phys Rev Lett* **72**, 1565-1568 (1994).

9. Emura S, Nakagawa T, Gonda S, Shimizu S. Raman-Spectra of Alxin1-Xas Grown by Molecular-Beam Epitaxy. *J Appl Phys* **62**, 4632-4634 (1987).

10. Wu J, *et al.* Unusual properties of the fundamental band gap of InN. *Appl Phys Lett* **80**, 3967-3969 (2002).

11. Fehse R, *et al.* Evidence for large monomolecular recombination contribution to threshold current in 1.3 mu m GaInNAs semiconductor lasers. *Electron Lett* **37**, 1518-1520 (2001).

12. Jin SR, Zheng YL, Li AZ. Characterization of photoluminescence intensity and efficiency of free excitons in semiconductor quantum well structures. *J Appl Phys* **82**, 3870-3873 (1997).

13. Manna S, Katiyar A, Aluguri R, Ray SK. Temperature dependent photoluminescence and electroluminescence characteristics of core-shell Ge-GeO2 nanowires. *J Phys D Appl Phys* **48**, (2015).

14. Sun XC, Liu JF, Kimerling LC, Michel J. Direct gap photoluminescence of n-type tensile-strained Ge-on-Si. *Appl Phys Lett* **95**, (2009).
